# The Essential Role Played by B Cells in Supporting Protective Immunity Against *Trichuris muris* Infection Is by Controlling the Th1/Th2 Balance in the Mesenteric Lymph Nodes and Depends on Host Genetic Background

**DOI:** 10.3389/fimmu.2019.02842

**Published:** 2019-12-10

**Authors:** Rinal Sahputra, Dominik Ruckerl, Kevin N. Couper, Werner Muller, Kathryn J. Else

**Affiliations:** Division of Infection, Immunity and Respiratory Medicine, Lydia Becker Institute for Immunology, The University of Manchester, Manchester, United Kingdom

**Keywords:** B cells, *Trichuris muris*, anti-CD20 mAb, Th2 immunity, IFN-γ

## Abstract

How B cells contribute to protective immunity against parasitic nematodes remains unclear, with their importance as accessory cells underexplored. In this study, anti-CD20 monoclonal antibody (α-CD20 mAb)-mediated depletion of B cells from C57BL/6 mice revealed an important role for B cells in supporting Th2 immune responses and thus expulsion of *Trichuris muris* (*T. muris*). C57BL/6 mice normally mount mixed Th1/Th2 immune responses to *T. muris* and expel the parasite by the third week post infection. However, B cell-depleted C57BL/6 had significantly reduced Th2-type cytokines post infection and failed to expel the parasite. IFN-γ production in the MLN of C57BL/6 mice receiving α-CD20 mAb treatment was not affected, collectively resulting in an overall change in Th1/Th2 balance in favor of Th1. Further, the expression of IFN-γ and IFN-γ-induced genes at the effector site, the gut, was significantly increased in the absence of B cells. Interestingly, and in complete contrast, BALB/c mice, which mount strongly polarized Th2 immune responses, rather than mixed Th1/Th2 immune responses, were still able to expel *T. muris* in the absence of B cells. We thus hypothesized that the B cell plays a critical role in enabling strong Th2 responses in the context of mixed Th1/Th2 settings, with the role becoming redundant in highly Th2 polarized environments. In support of this, neutralization of IFN-γ in B cell depleted C57BL/6 restored resistance against *T. muris* infection. Thus, our data suggest an important role of B cells in supporting Th2-type immune responses in mixed IFN-γ-rich Th1/Th2 settings.

## Introduction

Infecting over two billion people around the world, mostly in resource-limited countries, the ability of parasitic helminths to maintain long-standing chronic infections makes them a major health care issue (1). *Trichuris trichiura* (*T. trichiura*) is one of the most common gastrointestinal nematodes, infecting ~465 million people worldwide (2). In infected children, trichuriasis is strongly associated with malnutrition, growth stunting, and reduced educational performance, whereas in adults, it is related to anemia, reduced worker productivity, and, in women, low-birth-weight babies (3).

For decades, *Trichuris muris* in the mouse has provided a useful and relevant model system with which to explore immunity to *T. trichiura* in man due to their homology at the genomic and transcriptomic level (4, 5). *Trichuris* parasites secrete a heterogeneous array of molecules collectively referred to as the excretome/secretome (E/S), which can stimulate the host immune system (6, 7). Infection of mice with the intestinal nematode parasite *T. muris* drives polarized T helper cell (Th) responses, which associate with resistance (Th2) or susceptibility (Th1) (4). However, the key cellular contributions that support Th2 cell polarization during *T. muris* infection remain unclear. One of the cells thought to be important is the B cell. B cell function is not only related to antibody production, with B cells acting as antigen-presenting cells (APCs) (8–10) and as accessory cells, through their ability to secrete multiple cytokines (11).

Many studies have revealed the importance of CD4 T cells in mediating resistance against *T. muris* (12–14). In contrast, B cells and antibody were thought not to be important in mediating resistance to a primary *T. muris* infection (15–17). Arguing against this, however, *T. muris*-infected μMT mice on a C57BL6 background develop Th-1 type responses, resulting in susceptibility to *T. muris* (18). Furthermore, when these mice were treated with B cells from naive C57BL6 or with anti-IL-12 antibody, resistance to infection was restored (18). These data thus suggest that B cells are important in either inhibiting Th1 development or supporting Th2-type immune responses. However, given the importance of B cells in the development of lymph nodes and tissue organization (19, 20), data from μMT mice are difficult to interpret.

This study therefore investigated the role of B cells and antibodies in immunity to *T. muris* infection using α-CD20 mAb to deplete B cells from C57BL/6 and BALB/c mice. Adopting an α-CD20 mAb-mediated B cell approach allows for the depletion of CD19+ cells either prior to or post infection Further, it avoids the complicating consequences of B cell deficiency during embryonic development (21, 22). We demonstrate that B cells are important in the development and maintenance of the protective immune response to *T. muris*. Further, we show that this B cell dependency is only relevant in mixed Th1/Th2 IFN-γ rich settings, as seen in C57BL/6 mice, and is redundant in the highly Th2 polarized, IFN–γ-deficient environment of the BALB/c mouse post *T. muris* infection.

## Materials and Methods

### Animals

C57BL/6 and BALB/c mice were purchased from Envigo, UK, and were maintained in ventilated cages in the Biological Services Facilities (BSF) of the University of Manchester according to the UK Animals (Scientific Procedures) Act (1986). Male mice were housed in the facility at least 7 days prior to experimentation and were infected at 6–8 weeks old with *T. muris* by oral gavage.

For high-dose *T. muris* infection, ~3–4 ml of egg suspension was transferred to a universal tube and topped up with deionized water before centrifuging for 15 min at 2,000 g. Pelleted eggs were washed with deionized water and resuspended, and only embryonated eggs were counted. Eggs were concentrated or diluted with deionized water, depending on the egg count. Mice were then infected with 150 infective *T. muris* eggs in 200 μl by oral gavage at day 7 after α-CD20 mAb treatment or 14 days before α-CD20 mAb treatment.

### Maintenance of Parasite and Preparation of Egg Batches

All protocols to maintain the parasite were as previously described (21–24). The parasite was passaged through SCID mice that are susceptible to *T. muris* infection. SCID mice received a high dose of 150 infective *T.muris* eggs, and at day 35 post infection (p.i.), adult worms were collected from the large intestine. *T. muris* eggs from adult worms after overnight culture at 37°C were resuspended in 40 ml of deionized water and filtered through a 100-μm nylon sieve before transferring to a cell culture flask. To allow embryonation, eggs were stored in darkness at room temperature for ~8 weeks and then stored at 4°C. In order to establish the number of eggs required to establish around 100 worms, all egg batches were tested in SCID mice prior to experimental use, to determine the infectivity of each new batch of eggs. Thus, larvae were counted at around day 14 p.i. and the number of larvae counted was expressed as a % over the number of eggs given to determine the infectivity of the egg batch. Day 14 was chosen as the time point to assess egg batch infectivity as it is technically easier to count day 14 larvae than the smaller larvae that exist at earlier time points.

### Preparation of Excretory/Secretory (E/S) Products

The protocols to prepare the E/S products were as previously described (23, 24). Adult worms were pulled from the gut and transferred to a six-well plate containing 4 ml of warmed 5× pen/strep in RMPI 1640 medium. Plates were incubated in a moist humidity box for 4 h at 37°C to collect 4 h E/S. For overnight E/S, adult worms were then split into two wells containing fresh medium and incubated again in a humidity box at 37°C overnight. Supernatant from 4-h and overnight incubations was collected and centrifuged at 2,000 g for 15 min. All E/S supernatant was filter sterilized through a 0.2-μm syringe filter (Merk). E/S was concentrated using an Amicon Ultra-15 centrifugal filter unit (Millipore) by spinning at 3,000 g for 15 min at 4°C. E/S was dialyzed against PBS using Slide-A-Lyzer Dialysis Cassettes, 3.500 MWCO (Thermo Science) at 4°C. The protein concentration of E/S products was measured using the Nanodrop 1000 spectrophotometer (Thermo Fisher Science) and aliquoted before storing at −80°C.

### Quantification of *T. muris* Worms

During necropsy, the cecum and proximal colon were collected and stored at −20°C before analysis (22). Before worm count, the intestine was thawed at room temperature and cut longitudinally using blunt-ended scissors and the epithelium was scraped using curved forceps in a petri dish. Worms were counted blindly under a dissecting microscope (Leica S8 APO).

### B Cell Depletion and Infection

To assess the importance of B cells in worm expulsion in different strains, C57BL/6 and BALB/c mice were split into two groups of four to five mice: α-CD20 mAb and isotype control-treated mice. C57BL/6 or BALB/c mice were treated with 100 μg in 200 μl of PBS of α-CD20 mAb (5D2, Genentech) or isotype control (Rat IgG2a, Biolegend) by i.v. injection via the tail vein. Mice were infected with ~200 *T. muris* eggs at day 7 post injection. Mice were re-treated with α-CD20 mAb or isotype control at day 10 p.i. C57BL/6 mice were necropsied at day 35 p.i., while BALB/c mice were necropsied at day 42 p.i. To assess whether B cells are also important in maintaining Th2 immune responses during *T. muris* infection, C57BL/6 mice were treated with α-CD20 mAb or isotype control at day 14 and day 24 p.i. For anti-IFN-γ, C57BL/6 mice were split into four groups of five mice: isotype control of α-CD20 mAb + isotype control of α-IFN-γ mAb (rat Ig), α-CD20 mAb + isotype control of anti-IFN-γ mAb, isotype control of α-CD20 mAb + anti-IFN-γ mAb, and α-CD20 mAb + α-IFN-γ mAb.

### Preparation of Single-Cell Suspensions for Flow Cytometry

Mesenteric lymph nodes (MLNs), spleen, blood, and bone marrow were collected and prepared for fluorescence-activated cell sorting (FACS) staining. Lymph nodes and spleen were squeezed through a 70-μm nylon cell strainer (Fisher Scientific) and cells were pelleted by centrifugation at 1500 rpm for 5 min. The supernatant was removed and the pelleted cells were resuspended in 500 μl to 1 ml of Red Blood Cell Lysing Buffer Hybri-Max™ (Sigma-Aldrich) for 30 s to 1 min before adding 10 ml 1× PBS. Cells were pelleted by centrifugation at 1500 rpm for 5 min and resuspended in 1 ml of complete RPMI 1640 medium. Cells were counted on a CASY cell counter (Scharfe System).

Approximately 50 μl of blood was placed into a 1.5-ml eppendorf tube containing 50 μl of 0.5 M EDTA and stored on ice before analysis. Five hundred microliters of Red Blood Cell Lysing Buffer Hybri-Max™ (Sigma-Aldrich) was added, and samples were incubated for 5 min at room temperature. One milliliter of 1× PBS was added and cells were pelleted by centrifugation at 1,500 rpm for 5 min. Red blood cell lysis process was repeated twice before cells were resuspended in 1 ml of complete RPMI 1640 medium.

Femurs and tibias were collected at necropsy, and after removing any remaining tissue without damaging the bone integrity, the bone was placed on ice until ready to process. Bone was transferred to 70% ethanol for 2–3 min and then rinsed in three changes of 1× PBS. In a petri dish, both ends of bone were cut and the bone was flushed gently with 1× PBS using a 3-cc syringe and a 23-ga needle. To break up the clumps, the marrow was sucked up and was gently pushed back. Single-cell suspension was filtered through a 100-μm nylon cell strainer (Fisher Scientific) and cells were pelleted by centrifugation at 1500 rpm for 5 min and resuspended in 1 ml of 1× PBS. The supernatant was removed, and the pelleted cells were resuspended in 500 μl of Red Blood Cell Lysing Buffer Hybri-Max™ (Sigma-Aldrich) for 1 min before adding 10 ml of 1× PBS. Cells were pelleted by centrifugation at 1500 rpm for 5 min and resuspended in 1 ml of complete RPMI 1640 medium.

### Cell Surface Markers

Cells from MLNs, spleen, blood, and bone marrow were stained for live dead (Zombie UV, Biolegend) and Fc block (eBiosciences) prior to cell surface cellular markers staining. Samples were read on a BD LSR Fortessa flow cytometer (BD Biosciences), and data were analyzed using FlowJo X (Tree Star, Inc).

Cell surface markers: anti-B220 (RA3-6B2), anti-CD19 (6D5), anti-CD3ε (17A2), anti-CD4 (RM4.5), anti-CD8α (53-6.7), anti-CD279 (PD-1) (29F.1A12), anti-CD185/CXCR5 (L138D7), anti-CD93 (AA4.1), anti-CD25 (3C7), and anti-CD117/c-kit (2B8) were purchased from Biolegend. Anti-CD43 (S11), anti-CD103 (2E7), anti-CD11c (N418), anti-CD317/PDCA-1 (927), anti CD11b (M1/70), anti-CD64 (X54-5/7.1), anti-I-A/I-E (M5/114.15.2), and anti-CD23 (B3B4) were purchased from BD Biosciences. Anti-CD45 (30-F11), anti-ly6G (RB6-8C5), anti-NK.1 (PK136), and anti-Ter119 (Ter-119) were purchased from eBiosciences.

### Quantification of Parasite Specific IgG1, IgG2a/c, and IgM

To detect IgG1-, IgG2a/c-, and IgM-specific *T. muris*, an enzyme linked immunosorbant assay (ELISA) was completed. Blood was collected from mice at necropsy, and serum was isolated by centrifuging samples for 10 min at 15,000 g at room temperature. Ninety-six-well immunoGrade plates (BrandTech Scientific, Inc) were coated with 5 μg/ml *T. muris* E/S diluted in 0.05 M carbonate/bicarbonate buffer and incubated on plates overnight at 4°C. Plates were washed using a Skatron Scan Washer 500 (Molecular Devices, Norway) three times with 0.05% Tween 20 (Sigma) in PBS (PBS-T). Non-specific binding were blocked with 100 μl of 3% bovine serum albumin (BSA) (Melford Laboratories)/PBS at 37°C for 45 min. Plates were washed three times with PBS-T, and 50 μl of double diluted serum in PBS (1:20, 1:40, 1:80, 1:160, 1:320, 1:640, 1:1280, 1:2560) was added to plates and incubated for 60 min. Plates were washed three times with PBS-T and 50 μl of either biotinylated rat anti-mouse IgG1 (1:500, BD Bioscience), rat anti-mouse IgG2a/c (1:1,000, BD Bioscience), or rat anti-mouse IgM (1:500, BD Bioscience) added to wells and incubated for 60 min. Plates were then washed with PBS-T three times before the plates were incubated with 75 μl of Streptavidin peroxidase (1:1,000, Sigma) for 60 min. Plates were washed three times with PBS-T and then the substrate, 100 μl of 0.03% hydrogen peroxidase-activated ABTS (10% 2,2'azino 3-thyl benzthiazoline in 0.045 M citrate buffer), was added. Plates were read at 450 nm on a VersaMax Microplate reader (Molecular Devices).

### Cytokine Analysis

Cells were plated at a final concentration of 5 × 10^6^ cells/ml with 50 μg/ml 4 h E/S in a 96-well flat bottom plate. Restimulation of MLN cells with E/S products allows analyses of parasite-specific immune responses *ex vivo* and is a standard method (23). Leukocytes in the draining MLNs are primed *in vivo* by the parasite E/S, and restimulation of these cells *in vitro* with E/S stimulates them to secrete the profile of cytokines that they have been primed to secrete *in vivo* in an antigen-specific way. Cells were incubated for 48 h at 37°C and 5% CO_2_. To collect the supernatant, plates were centrifuged at 1,400 g for 5 min and the supernatant was stored at −20°C.

The cytokines IL-4, IL-5, IL-6, IL-9, IL-10, IL-17, IL-13, TNF, and IFN-γ were detected in supernatant by cytometric bead assay (CBA). 12.5 μl of supernatant from *T. muris* E/S-stimulated cells was added to a 96-well round bottom plate. A capture bead cocktail (BD Bioscience), containing beads for each cytokine, was diluted in capture diluent (BD Bioscience) and 12.5 μl was added to each well before incubating on a rocker at room temperature for 1 h. 12.5 μl of detection beads (BD Bioscience), diluted in detection reagent (BD Bioscience), was added to each well and incubated again on a rocker at room temperature for 1 h. Plates were washed and resuspended in 70 μl of wash buffer (BD Bioscience). Cytokines were measured on a MACSQuant Analyser (Miltenyi Biotec) and analyzed using the FCAP array software in reference to a standard curve.

### Histology

Sections of proximal colon were fixed at room temperature in 10% neutral buffered formalin containing 0.9% sodium chloride, 2% glacial acetic acid, and 0.05% alkyltrimethyl-ammonium bromide before they were processed and embedded in paraffin wax as previously described (24). Serial sections were cut at 5 μm thick transparietally and stained with periodic acid-Schiff's protocol. Slides were placed in citroclear (T.C.S Bioscience Ltd.) twice for 5 min, rehydrated through a decreasing concentration of alcohol solution at 100, 90, 70, and 50% for 1 min each. Slides were then washed with distilled water for 1 min and stained with 1% periodic acid (1 g of periodic acid in 100 ml of distilled water) for 5 min. Slides were rinsed with distilled water, tap water, and distilled water again for 1 min each. Slides were stained with Schiff's reagent (Sigma-Aldrich) for 10 min, rinsed with distilled water, tap water, and distilled water for 1 min each. Slides were stained with Mayer's hematoxylin (Sigma-Aldrich) for 30 s, placed under running water for 5 min, and then rinsed with distilled water for 1 min. Slides were dehydrated by placing through an increasing alcohol concentration at 50, 70, 90, and 100% for 30 s in each concentration. Finally, slides were dipped in citroclear (T.C.S Bioscience Ltd.) twice for 4 min and 3 min and mounted with DPX mounting medium (Fisher Scientific) and left to dry. The number of goblet cells per mouse was calculated from the average of three sets of counts over 20 crypt units for each mouse. For immunohistochemical staining, sections of proximal colon were deparaffinized, and rehydrated through decreasing alcohol concentration. For antigen retrieval, the tissues were treated with 10 mM citrate buffer (pH 6.0) at 95°C for 30 min in water bath. After endogenous peroxidase activity was extinguished with 3% H_2_O_2_ for 10 min, the tissues were blocked with normal rat serum for 60 min at room temperature. The tissues were stained with biotinylated rat anti-mouse CD45R/B220 (5 μg/ml, RA3-6B2; BD-Biosciences) overnight at 4°C. Negative controls consisted of sections incubated with biotinylated rat IgG2a isotype control (Biolegend). The tissues were stained with ABC (avidin-biotin-peroxidase) kit (Vector Laboratories), followed by DAB (3,3′-diaminobenzidin chromagen) kit (Vector Laboratories) according to the manufacturer's instruction. The tissues were counterstained with a drop of Harris's hematoxylin solution for 1 min and mounted in Aquamount aqueous mounting medium (BDH-Merck). Images were acquired using a [20×/0.80 Plan Apo] objective using the 3D Histech Pannoramic 250 Flash II slide scanner.

### qPCR

The production of *Ifn*γ, *Il13, Cxcl10, Tgtp, Relm*β, and *Il17a* mRNA in the cecal mucosa was examined using RT-PCR. The total RNA was extracted from cecal tips using TRIzol reagent (Invitrogen) and stored at −80°C prior to use. cDNA was synthesized from 2 μg of RNA using the High-Capacity cDNA Reverse Transcription Kits (Life Technologies) containing recombinant moloney murine leukemia virus reverse transcriptase (MultiScribe™ RT, Life Technologies). After cDNA synthesis, a Brilliant III Ultra-Fast SYBR® Green QPCR Master Mix (Agilent Technologies) was used for RT-PCR. The primer sequences used were *Ifn*γ: 5-TGAGCTCATTGAATGCTTGG-3 and 5-ACAGCAAGGCGAAAAAGGAT-3; *Il13*: 5-CACACTCCATACCATGCTGC-3 and 5-TGTGTCTCTCCCTCTGACCC-3; *Tgtp*: 5-GGCCAGTTGTGCATCATTTTC-3 and 5-TGGGACCACTAACTTCACACC-3; *Cxcl10*: 5-CGCGGATCCAGGAGATCTTTTAGACATTTC-3 and 5-CGCAAGCTTCGCGAGACATTCCTCAATTGC-3; *Il17a*: 5-GGATTTCGTGGGATTGTGAT-3 and 5-TGGGAAGACGTCATTGGTGT-3; *Eef* : 5-TGTCAGTCATCGCCCATGTG-3 and 5-CATCCTTGCGAGTGTCAGTGA-3. All primers were purchased from Eurofins Biogenomic.

### Statistical Analysis

Statistical analysis was performed using Prism4 (GraphPad Software Inc., La Jolla, CA). The significant differences between two groups (*p* < 0.05) were analyzed with the *t* test or Mann Whitney test, depending on the *n*-size and the distribution of samples. For multiple groups, the significant differences were analyzed by ANOVA.

### Ethics Statement

All experiments were approved by The University of Manchester Local Animal Welfare and Ethical Review Body and were performed in accordance with the UK Home Office Animals (Scientific Procedures) Act 1986, under the Home Office project license number 70/8127.

## Results

### C57BL/6 Mice Treated With α-CD20 mAb Fail to Expel *T. muris* by d35 p.i., Correlating With an Absence of Class-Switched Antibodies and a Significant Decrease in Th2 Cytokines in the MLNs

**Figure 1:**
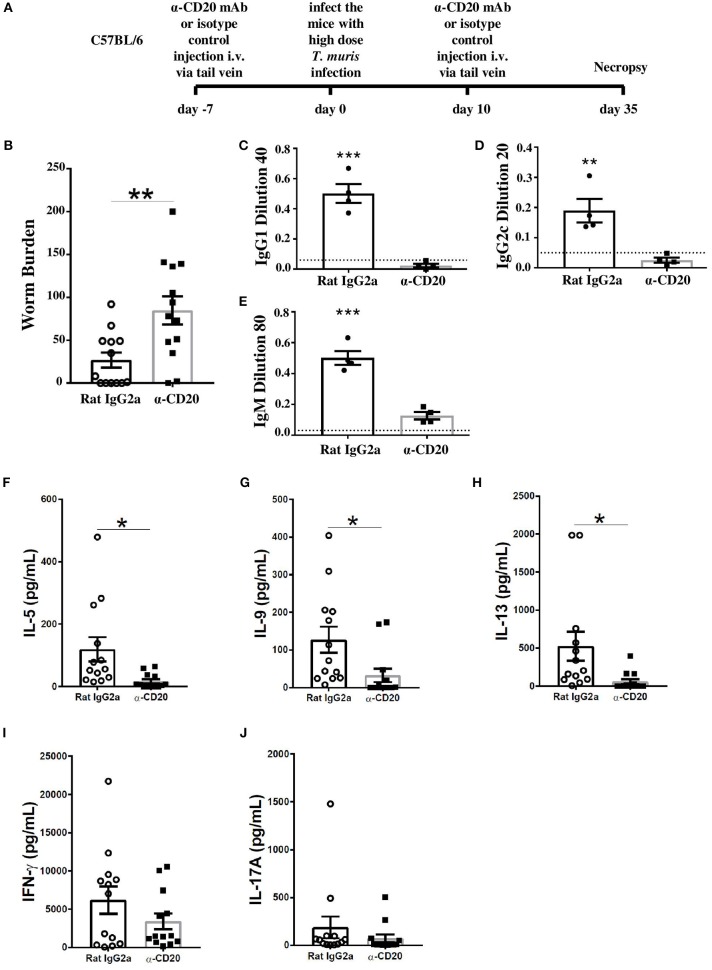
B cell-depleted C57BL6 mice were susceptible to *Trichuris muris (T. muris)*, correlating with an absence of class-switched antibodies, an unchanged Th1 response, and a significant decrease in Th2 cytokines. C57BL/6 mice were treated with α-CD20 mAb or Rat IgG2a isotype control at 100 μg in 200 μl of PBS by i.v. injection via tail vein prior to infection. Mice were infected with 150 infective *T. muris* eggs. Mice were necropsied at day 35 p.i. **(A)** Diagram of experimental design. **(B)** Worm burdens were assessed blindly after necropsy. **(C–E)** Sera were analyzed using ELISA for parasite specific antibodies. **(F–J)** MLN cells were re-stimulated with parasite E/S antigen for 48 h and cytokines in supernatants were determined using cytokine bead array (CBA). Data show mean ± SEM, pooled from three independent experiments **(B,F–J)**, representative of three independent experiments **(C–E)**, males, *p < 0.05, **p < 0.01, ***p < 0.001, Student's *t* test.

To investigate whether B cells are important in resistance to *T. muris* during a primary infection of C57BL/6 mice, mice were treated with α-CD20 mAb 7 days prior to infection and again at 10 days p.i., given that a single α-CD20 mAb treatment failed to ablate B cells for the full duration of *T. muris* infection (Supplementary Figure 1). Chronic infection, characterized by persisting adult stage parasites from day 32 p.i., defines susceptibility (16). Thus, necropsies were performed beyond this time point. The experimental design is shown in Figure 1A. Previous studies have shown that C57BL/6 mice take up to 35 days to completely expel the parasite (25). As shown in Figure 1B, C57BL/6 depleted of B cells were significantly more susceptible to infection than control-treated mice.

It has previously been shown that IgG1 antibodies are associated with resistance to *T. muris* infection, while IgG2a/c antibodies correlate with susceptibility (23). Therefore, the levels of parasite-specific antibodies in the sera of α-CD20 mAb and isotype control-treated mice were compared. Mice depleted of B cells using α-CD20 mAb failed to secrete IgG1 and IgG2c parasite specific antibodies, in contrast to control-treated mice (Figures 1C,D). Further, the levels of *T. muris*-specific IgM antibodies in the sera of α-CD20 mAb-treated mice were significantly lower than in the sera of the isotype control-treated mice (Figure 1E). These effects of B cell depletion on antigen-specific IgG and IgM antibodies are consistent with reports in other model systems (26).

In common with other nematode parasites, hosts resistant to *T. muris* mount Th2 immune responses characterized by the production of IL-4, IL-5, IL-9, and IL-13; in contrast, mice susceptible to *T. muris* mount a Th-1 type response, dominated by the release of IFN-γ (27–29). In order to assess changes in the quality of the Th cell response, cells from the local draining lymph nodes, MLNs, were re-stimulated *in vitro* with parasite E/S. Parasite-specific stimulation of MLN cells using parasite E/S has been used extensively before by ourselves and other groups (25, 30). E/S re-stimulation of MLN cells from uninfected mice made very low (pg/ml) levels of cytokines as shown in Supplementary Figure 2. B cell-depleted infected mice on a C57BL/6 background produced significantly lower Th2 cytokines, including IL-5, IL-9, and IL-13 compared to infected mice treated with the isotype control (Figures 1F–H). Interestingly, the production of IFN-γ and IL-17 was similar in both groups (Figures 1I,J) rather than increased in B cell-depleted mice, suggesting that the MLN B cell promotes Th2 responses rather than directly down-regulating Th1 responses.

### The Expression of IFN-γ and IFN-γ-Induced Genes in the Gut of Anti-CD20-Treated Mice Was Significantly Increased

**Figure 2:**
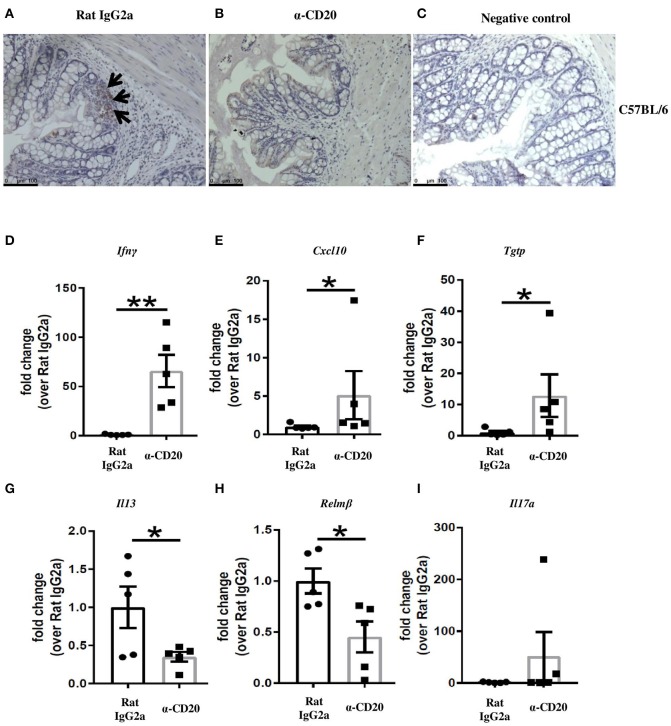
Treatment with α-CD20 mAb depleted B cells in the gut and increased the expression of IFN-γ and IFN-γ-induced genes. C57BL/6 mice were treated with α-CD20 mAb or Rat IgG2a isotype control at 100 μg in 200 μl PBS by i.v. injection via the tail vein. Mice were infected with 150 infective *T. muris* eggs at day 7 post injection. Mice were re-treated with α-CD20 mAb or isotype control at 100 μg in 200 μl of PBS by i.v. injection via the tail vein at day 10 p.i. Mice were necropsied at day 35 p.i. B cells in the gut of α-CD20 mAb and isotype-treated mice were assessed by staining the gut with biotinylated rat anti-mouse CD45R/B220 overnight and then with ABC (avidin-biotin-peroxidase) kit (Vector Laboratories), followed by DAB (3,3′-diaminobenzidin chromagen) kit according to the manufacturer's instruction. **(A,B)** Representative of B220 staining on the gut of isotype control and α-CD20 treated mice, respectively. **(C)** Negative controls consisted of sections incubated with biotinylated rat IgG2a isotype control. **(D–I)** Gene expression of *Ifn*γ, *Cxcl10, Tgtp, Il13, Relm*β, and *Il17a* in the cecal mucosa was examined using RT-PCR. Data show mean ± SEM, from one experiment, *n* = 5, males, *p < 0.05, **p < 0.01, Mann–Whitney test.

Although B cell depletion did not alter IFN-γ production in MLNs, we asked whether the overall shift in Th1/Th2 balance in the MLNs toward Th1 in mice receiving α-CD20 mAb would alter the expression of IFN-γ and IFN-γ-induced genes locally in the gut. Thus, IFN-γ and IFN-γ responsive genes were analyzed by qPCR. Figures 2A–C show a loss of B cells in the cecal tissue after α-CD20 mAb treatment. Our data revealed that the expression of IFN-γ (Figure 2D) and IFN-γ-induced genes, including *Cxcl10* and *Tgtp* (Figures 2E,F) in the gut of α-CD20-treated mice, were significantly increased compared to isotype control-treated mice. Furthermore, the expression of IL-13 and Relm-β in the gut of mice receiving α-CD20 treatment was significantly reduced (Figures 2G,H). IL-17a levels were not altered in the absence of B cells (Figure 2I). In keeping with an increased local gut Th1 environment, goblet cell numbers were reduced (Supplementary Figures 3A–C) and gut pathology was increased (Supplementary Figures 3D,E) in B cell-depleted mice. These data suggest that B cell depletion alters the Th1/Th2 balance in the MLN in favor of Th1 (Th2 reduced; Th1 unchanged). The altered Th1/Th2 balance presents itself locally in the gut as an increased Th1 environment and failure to eliminate the parasite.

### B Cell Depletion on Day 14 and Day 24 p.i. Also Inhibited Worm Expulsion

**Figure 3:**
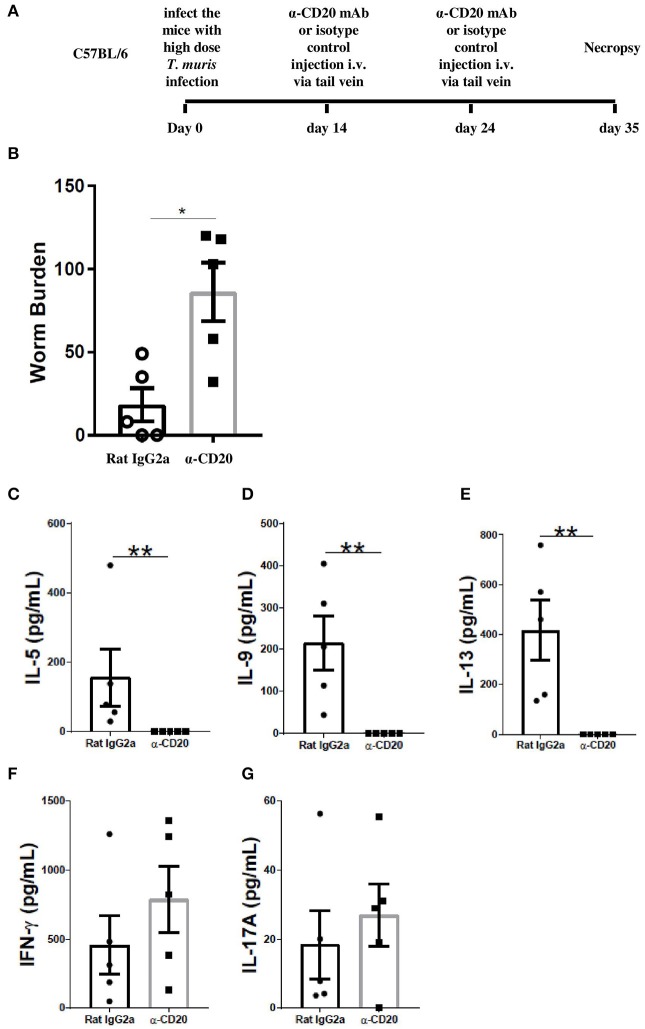
B cell depletion at day 14 p.i. and day 24 p.i. also led to susceptibility to *T. muris* infection. C57BL/6 mice were infected with 150 infective *T. muris* eggs and then mice were treated with α-CD20 mAb or Rat IgG2a isotype control at 100 μg in 200 μl of PBS by i.v. injection via the tail vein at day 14 p.i. and day 24 p.i. Mice were necropsied on day 35 p.i. **(A)** Diagram of the experiment design for B cell depletion 2 weeks p.i. **(B)** Worm burdens were assessed blindly after necropsy. **(C–G)** MLN cells were re-stimulated with parasite E/S antigen for 48 h and cytokines in supernatants were determined using cytokine bead array (CBA). Data show mean ± SEM from one experiment, *n* = 5, males, *p < 0.05, **p < 0.01, Mann–Whitney test.

Previous studies on the expulsion kinetics of *T. muris* have shown that the onset of worm expulsion occurs after day 12 p.i., with T cell activation occurring after the first 7 days of infection (31). Therefore, B cells were depleted 2 weeks post *T. muris* infection to see if the B cell contributed to the maintenance of an established Th cell response. The experimental design is shown in Figure 3A. Surprisingly, the depletion of B cells from day 14 p.i. (α-CD20 mAb given on day 14 p.i. and day 24 p.i.) also impaired worm expulsion with significantly more parasites present at day 35 p.i. than seen in control-treated mice (Figure 3B). Th2-type cytokines, including IL-5, IL-9, and IL-13, were significantly reduced (Figures 3C–E), while IFN-γ and IL-17A remained the same between groups (Figures 3F,G).

### Anti-IFN-γ Treatment Partially Restored MLN IL-13 Production in B Cell-Depleted Mice and Rescued Worm Expulsion in the Absence of Antibody

**Figure 4:**
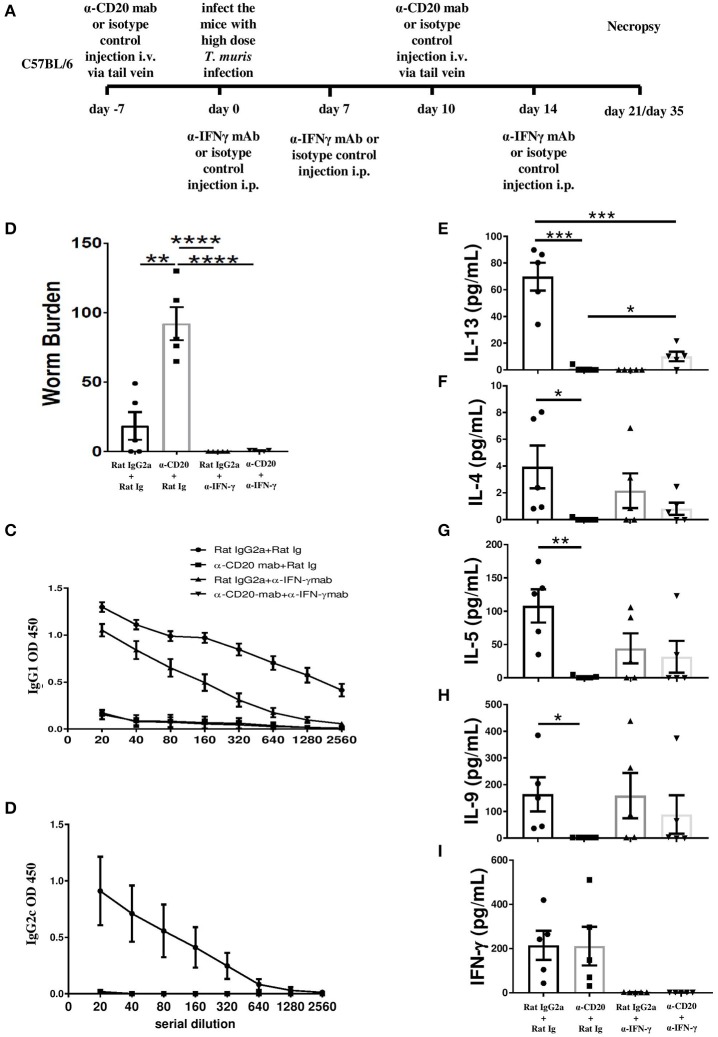
Anti-IFN-γ treatment restored resistance to *T. muris* infection and partially rescued the IL-13 response in B cell-depleted mice in the absence of *T. muris-*specific IgG1 antibodies. C57BL/6 mice were treated with α-CD20 mAb or isotype control at 100 μg in 200 μl of PBS by i.v. injection via the tail vein. Mice were infected with ~150 infective *T. muris* eggs at day 7 post injection. Mice were re-treated with α-CD20 mAb or isotype control at 100 μg in 200 μl of PBS i.v. injection via tail vein on day 10 p.i. One milligram of α-IFN-γ antibody or Rat Ig (as a control) was given on day 0, day 7, and day 14 p.i. Mice were necropsied at day 21 p.i. and day 35 p.i. **(A)** Diagram of the experimental design. **(B)** Worm burdens were assessed blindly after necropsy at day 35 p.i. **(C,D)** *T. muris-*specific IgG1 and IgG2c antibodies in the sera by day 21 p.i., respectively. **(E–I)** Cytokine analysis of re-stimulated MLN cells at day 21 p.i. Data show mean ± SEM, from one experiment, *n* = 5, males, *p < 0.05, **p < 0.01, ***p < 0.001, ****p < 0.0001, Mann–Whitney test.

In order to investigate whether the impaired resistance to infection in the absence of B cells was context dependent, B cell-depleted mice were injected with anti-IFN-γ. The experimental design for anti-IFN-γ injection plus B cell depletion is shown in Figure 4A. As shown in Figure 4B, anti-IFN-γ treatment restored resistance to *T. muris* in B cell-depleted mice, suggesting that the important role played by B cells in promoting Th2 responses is only relevant in the context of mixed Th1/Th2 settings.

As expected, blocking IFN-γ in the absence of B cells did not rescue the *T. muris*-specific IgG1 or IgG2a/c response (Figure 4C). *T. muris*-specific IgG2a/c antibodies were not detected in the sera of anti-IFN-γ-treated mice with intact B cells (Figure 4D), consistent with a highly polarized Th2 immune response. In contrast, the isotype control mice produced both IgG1 and IgG2a/c antibodies (Figures 4C,D). Consistent with our previous data, mice treated with α-CD20 mAb produced significantly lower Th2 cytokines, such as IL-13, IL-4, IL-5, and IL-9 compared to isotype control-treated mice (Figures 4E–H) with the Th1 response unaffected (Figure 4I). Importantly, blocking IFN-γ significantly increased MLN IL-13 production in mice depleted of B cells (Figure 4E). Although levels were still significantly lower than in isotype control-treated mice without any IFN-γ, partial restoration of Th2 immune response was sufficient to render animals resistant to infection. Although no significant difference was noted in the production of other Th2-type cytokines, there were trends toward increased *T. muris*-specific IL-4, IL-5, and IL-9 after anti-IFN-γ treatment in B cell-depleted mice, compared to mice depleted of B cells alone (Figures 4F–H).

### In the Absence of B Cell, Neutralization of IFN-γ Significantly Reduced IFN-γ and IFN-γ-Induced Gene Expression in the Intestine and Restored the Expression of IL-13

**Figure 5:**
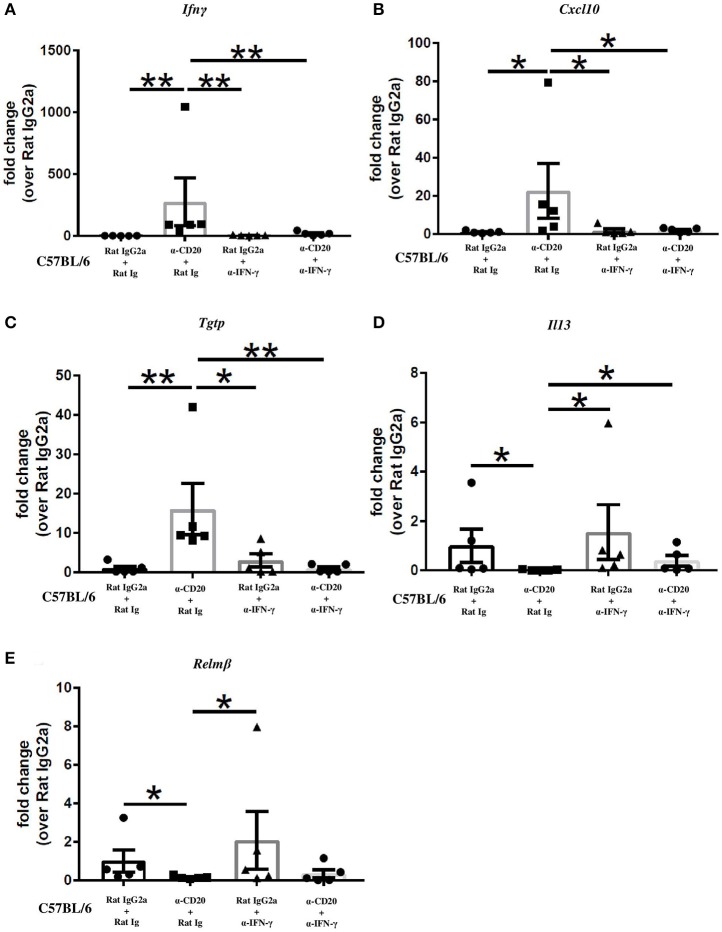
Neutralization of IFN-γ significantly reduced IFN-γ and IFN-γ-induced gene expression and restored the expression of IL-13 in the gut in the absence of B cells. C57BL/6 mice were treated with α-CD20 mAb or isotype control at 100 μg in 200 μl of PBS by i.v. injection via the tail vein. Mice were infected with 150 infective *T. muris* eggs at day 7 post injection. Mice were re-treated with α-CD20 mAb or isotype control at 100 μg in 200 μl of PBS i.v. injection via tail vein on day 10 p.i. One milligram of α-IFN-γ antibody or Rat Ig (as a control) was given on day 0, day 7, and day 14 p.i. Mice were necropsied at day 35 p.i. **(A–E)** Gene expression of *Ifn*γ, *Cxcl10, Tgtp, Il13*, and *Relm*β in the cecal mucosa was examined using RT-PCR. Data show mean ± SEM, from one experiment, *n* = 5, males, *p < 0.05, **p < 0.01, Mann–Whitney test.

Given that B cell depletion increased IFN-γ and IFN-γ-induced gene expression in the gut and at the same time reduced the expression of the Th2 markers, IL-13 and Relm-β, we asked whether α-IFN-γ would reverse those effects. In IFN-γ-depleted mice also depleted of B cells, intestinal expression of IFN-γ, and IFN-γ-induced genes was equivalent to that seen in isotype control-treated mice (Figures 5A–C) and significantly lower than seen in mice treated with α-CD20 mAb alone. The expression of IL-13 was significantly increased compared to α-CD20-treated mice (Figure 5D). Interestingly, gene expression of Relm-β in the gut of α-CD20 mAb treated mice was not significantly increased in the absence of IFN-γ (Figure 5E), suggesting that the presence of B cells is required for Relm-β production in the gut even in Th2 polarized environments.

### B Cells Are Essential in Supporting Th2 Immune Responses Against *T. muris* Only in an IFN-γ-Rich Environment

**Figure 6:**
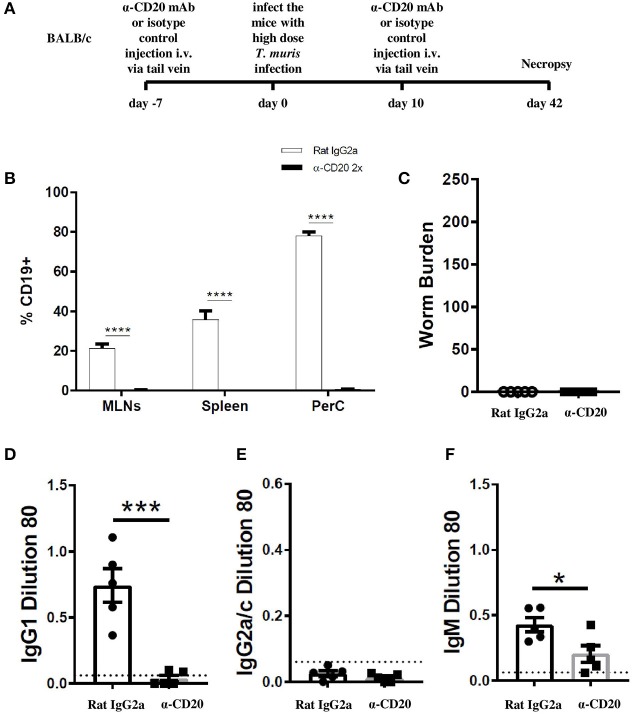
BALB/c mice were able to expel *T. muris* in the absence of B cells. BALB/c mice were treated with α-CD20 mAb or Rat IgG2a isotype control at 100 μg in 200 μl of PBS by i.v. injection via tail vein prior to infection. Mice were infected with 150 infective *T. muris* eggs and were necropsied at day 42 p.i. **(A)** Diagram of experimental design. **(B)** CD19+ cells were assessed in MLNs, spleen, and peritoneal cavity (PerC) using flow cytometry. **(C)** Worm burdens were assessed blindly after necropsy. **(D–F)** Sera were analyzed using ELISA for parasite-specific antibodies. Data show mean ± SEM, from one experiment, *n* = 5, males. *p < 0.05, ***p < 0.001, ****p < 0.0001, Mann–Whitney test.

In the current study, we found that when C57BL/6 mice were depleted of IFN-γ, the immune response became more highly polarized toward Th2 mice. In this more polarized Th2 environment, the depletion of B cells did not prevent worm expulsion. These data suggest that the important role played by B cells in supporting Th2 immune responses against *T. muris* is only necessary in IFN-γ-rich environments. Thus, B cells were depleted from BALB/c mice, which are naturally very resistant to *T. muris* and mount highly polarized Th2 immune responses (25). The experimental design is shown in Figure 6A. CD19+ cells in the MLN and spleen of BALB/c mice were not detected after α-CD20 mAb treatment (Figure 6B). In complete contrast to B cell-depleted C57BL/6 mice, BALB/c mice were still able to expel the parasite in the absence of B cells (Figure 6C). IgG1 and IgG2a/c parasite-specific antibodies were undetectable in B cell-depleted mice (Figures 6D,E), and levels of *T. muris*-specific IgM were significantly lower than in the sera of isotype control-treated mice (Figure 6F). As shown previously (25, 32), isotype control-treated BALB/c mice mounted strong parasite-specific IgG1 responses but did not secrete *T. muris*-specific IgG2a/c (Figures 6D,E), indicative of a highly polarized Th2 immune response.

## Discussion

*Trichuris muris* infection drives different Th cell responses in different strains of mice (28). BALB/c, BALB/k, and NIH mice are very resistant to *T. muris* infection, expelling the worms around day 18 p.i. C57BL/6 and C57BL/10 mice are also resistant to infection, but expel the worms more slowly, between day 18 and 35 p.i. (33). In contrast, AKR mice fail to expel the parasite and develop chronic infections (34). Susceptible hosts mount predominantly Th-1 immune responses associated with the presence of IFN-γ and IL-12, while very resistant host strains mount strong Th-2 type polarized immune response characterized by cytokines such as IL-4, IL-5, IL-9, and IL-13, and very low levels of IFN-γ. The characteristic of the Th cell response in C57BL/6 mice is less clearly polarized with the slower expulsion kinetic associated with a mixed Th1/Th2 phenotype and presence of IgG1 and IgG2c. Despite a good understanding of the T helper cell response during *T. muris* infection, the key cellular contributions that support T helper cell polarization are still not well understood, or how genetic background impinges on this.

This study aimed to investigate the role of B cells in immunity to *T. muris* during primary infection by using α-CD20 mAb to deplete B cells. In order to understand whether the contribution of the B cell varied with the nature of the protective immune response, we used mice of two different genetic backgrounds: C57BL/6 and BALB/c. CD20 is specifically expressed on the surface of B cells from the pre-B cells stage to immature B cells, but then disappear when B cells differentiate to plasma cells (35). Adding antibody against CD20 inhibits the progression of B cells from the G1 phase into S/G2+ M stages (36), resulting in the inhibition of B cell differentiation, antibody production, and inducing B cell apoptosis (37). Using anti-CD20-mediated depletion of B cells, antigen-specific class-switched antibodies are ablated and IgM antibodies are significantly reduced (38, 39). These findings are in keeping with the finding of the current study and enable an assessment of the importance of parasite-specific IgG1 antibodies in the expulsion of *T. muris*.

Our data suggest that antibody is not essential for the expulsion of *T. muris*, with both B cell-depleted BALB/c mice and B cell-depleted C57BL/6 mice also depleted of IFN-γ, able to clear infection. Unlike the C57BL/6 mouse, the BALB/c mouse is highly polarized toward Th2 post *T. muris* infection, with no IFN-γ made, as shown previously (25). In keeping with this, we have used IgG2a/c as a sensitive readout of any *in vivo* IFN-γ. Normally, IgG antibody isotype levels continue to rise well after worm expulsion (34). Thus, if any IFN-γ had been present *in vivo* in the host, we would detect this best at later time points post infection. In our data, we show that even after 42 days p.i., there is no IgG2a/c (but a strong IgG1 response). Adult *T. muris* parasites are present at any time point after 32 days of infection if the host has failed to expel the infection. Therefore, for the BALB/c experiments, we use day 42 to evidence the presence of IgG1, the lack of IgG2a/c, and worm clearance.

Antibody-independent expulsion of *T. muris* has been previously evidenced by the fact that primed CD4+ T cell transfer to SCID mice is sufficient to support worm expulsion in the absence of antibody (17). These data also provide new evidence that the mechanism of immunity to *T. muris* in BALB/c mice is entirely B cell independent. However, our data do identify an important and significant antibody-independent role for the B cell in promoting immunity to infection in mouse strains, such as C57BL/6, which are not highly polarized toward a Th2 immune response. Thus, the importance of the B cell in resistance to infection is dependent on genetic background and represents a key consideration when interpreting data from other experimental models. Further, given that protective immunity is lost when B cells are depleted both throughout the time course of infection and after the first 2 weeks of infection, our data suggest that the role played by the B cell is as an accessory cell supporting both the development and maintenance of Th2 immune responses.

Previous study had revealed that B cell depletion impaired the development of Th2 responses in mice infected with *Heligmosomoides polygyrus* (40). The importance of B cells in immunity to *T. muris* has previously been proposed using μMT mice (18) on a C57BL/6 background, which are susceptible to infection unless Th1 responses are inhibited using anti-IL-12. These data are in keeping with the current study supporting a role for B cells as accessory cells promoting and maintaining Th2 responses rather than as antibody producers.

More broadly, the role of B cells in immunity to gastrointestinal nematodes in general has been debated at length (41–44). Proposed mechanisms include production of antibody (45) and, in keeping with the current study, promoting, and maintaining of primary and memory Th2 cells (46, 47) via their ability to produce cytokines, especially IL-4 (41, 48, 49), and/or by expressing co-stimulatory molecules, including OX40L and CD40 (50, 51). A variety of experimental approaches have been used including the use of transgenic B cell-deficient mice (18, 45, 46, 52), FcγR deficiency (53, 54), passive immunization (18, 55, 56), and maternal antibody transfer (42, 57), and a variety of conclusions have been drawn. IgG1 antibodies were shown to be essential for worm expulsion against *H. polygyrus* infection based on previous studies using AID mice, which retain a secretory IgM response, on a C57BL/6 genetic background (45). Antibody was shown to be essential for worm expulsion against *H. polygyrus* infection via antibody-dependent cell-mediated cytotoxic (ADCC) mechanisms (57). However, ADCC mechanisms do not play a critical role in immunity to *T. muris* infection as FcγR deficiency mice are still able to expel the parasite (54).

Given that B cell depletion did not affect T cell-derived IFN-γ production in the MLN, while the Th2 response was reduced, and that the depletion of IFN-γ reversed the effect of B cell depletion in the context of worm expulsion, our data suggest that the B cell is important in promoting Th2 immune responses only in mixed Th1/Th2 settings.

Collectively, we present a study revealing that the important role played by the B cell in promoting resistance to *T. muris* infection in mixed Th1/Th2 cytokine settings is in supporting the development and maintenance of the Th2 immune response, rather than directly affecting the Th1 response. Further, the role played by the B cell is not related to antibody production. Thus, without B cells, MLN Th2 responses are significantly reduced with Th1 responses unchanged; thus, the Th1/Th2 balance in the MLN shifts toward Th1. The Th1-dominated response manifests itself locally at the effector site as elevations in IFN-γ and IFN-γ-responsive genes. Current research is focusing on identifying the signals B cells provide to support Th2 responses in mixed Th1/Th2 settings. Importantly, the essential role played by the B cell in the protective immune response varied with genetic background and the degree of T helper cell polarization of the host. Thus, if IFN-γ is depleted from mixed Th1/Th2 settings, or the Th2 immune response is dominant, the B cell becomes redundant in supporting the Th2 protective immune response.

## Data Availability Statement

All datasets generated for this study are included in the article/Supplementary Material.

## Ethics Statement

The animal study was reviewed and approved by The University of Manchester Local Animal Welfare and Ethical Review Body.

## Author Contributions

RS designed and performed experiments, analyzed data, and wrote the manuscript. KE designed experiments, analyzed data, and wrote the manuscript. KC, DR, and WM provided input for interpretation and edited the manuscript.

### Conflict of Interest

The authors declare that the research was conducted in the absence of any commercial or financial relationships that could be construed as a potential conflict of interest.

## References

[1] Hotez PJ Brindley PJ Bethony JM King CH Pearce EJ Jacobson J. Helminth infections: the great neglected tropical diseases. J Clin Invest. (2008) 118:1311–21. 10.1172/JCI34261 18382743 PMC2276811

[2] Zhan B Beaumier CM Briggs N Jones KM Keegan BP Bottazzi ME. Advancing a multivalent'Pan-anthelmintic'vaccine against soil-transmitted nematode infections. Exp Rev Vacc. (2014) 13:321–31. 10.1586/14760584.2014.872035 24392641 PMC3934375

[3] Bethony J Brooker S Albonico M Geiger SM Loukas A Diemert D. Soil-transmitted helminth infections: ascariasis, trichuriasis, and hookworm. Lancet. (2006) 367:1521–32. 10.1016/S0140-6736(06)68653-4 16679166

[4] Klementowicz JE Travis MA Grencis RK. *Trichuris muris*: a model of gastrointestinal parasite infection. Semin. Immunopathol. (2012) 34:815–28. 10.1007/s00281-012-0348-2 23053395 PMC3496546

[5] Foth BJ Tsai IJ Reid AJ Bancroft AJ Nichol S Tracey A. Whipworm genome and dual-species transcriptome analyses provide molecular insights into an intimate host-parasite interaction. Nat Genet. (2014) 46:693. 10.1038/ng.3010 24929830 PMC5012510

[6] Eichenberger RM Ryan S Jones L Buitrago G Polster R Montes de Oca M. Hookworm secreted extracellular vesicles interact with host cells and prevent inducible colitis in mice. Front Immunol. (2018) 9:850. 10.3389/fimmu.2018.00850 29760697 PMC5936971

[7] Bancroft AJ Levy CW Jowitt TA Hayes KS Thompson S Mckenzie EA. The major secreted protein of the whipworm parasite tethers to matrix and inhibits interleukin-13 function. Nat Commun. (2019) 10:2344. 10.1038/s41467-019-09996-z 31138806 PMC6538607

[8] Constant S Schweitzer N West J Ranney P Bottomly K. B lymphocytes can be competent antigen-presenting cells for priming CD4+ T cells to protein antigens *in vivo*. J Immunol. (1995) 155:3734–41. 7561077

[9] Liu Y Wu Y Ramarathinam L Guo Y Huszar D Trounstine M. Gene-targeted B-deficient mice reveal a critical role for B cells in the CD4 T cell response. Int Immunol. (1995) 7:1353–62. 10.1093/intimm/7.8.1353 7495742

[10] Magee CN Boenisch O Najafian N. The role of costimulatory molecules in directing the functional differentiation of alloreactive T helper cells. Am J Transplantat. (2012) 12:2588–600. 10.1111/j.1600-6143.2012.04180.x 22759274 PMC3459149

[11] Lund FE Garvy BA Randall TD Harris DP. Regulatory roles for cytokine-producing B cells in infection and autoimmune disease. Curr Dir Autoimmun. (2005) 8:25–54. 10.1159/000082086 15564716

[12] Betts C. J. Deschoolmeester M. L. Else K. J. (2000). *Trichuris muris*: CD4+ T cell-mediated protection in reconstituted SCID mice. Parasitology 121:631–7. 10.1017/S0031182000006740 11155934

[13] Little MC Bell LV Cliffe LJ Else KJ. The characterization of intraepithelial lymphocytes, lamina propria leukocytes, and isolated lymphoid follicles in the large intestine of mice infected with the intestinal nematode parasite *Trichuris muris*. J Immunol. (2005) 175:6713–22. 10.4049/jimmunol.175.10.6713 16272327

[14] Svensson M Russell K Mack M Else KJ CD4+ T-cell localization to the large intestinal mucosa during *Trichuris muris* infection is mediated by Gαi-coupled receptors but is CCR6-and CXCR3-independent. Immunology. (2010) 129:257–67. 10.1111/j.1365-2567.2009.03178.x 19824922 PMC2814467

[15] Yoichi I The absence of resistance in congenitally athymic nude mice toward infection with the intestinal nematode, Trichuris muris: resistance restored by lymphoid cell transfer. Int J Parasitol. (1991) 21:65–9. 10.1016/0020-7519(91)90121-M 2040569

[16] Koyama K Tamauchi H Ito Y. The role of CD4+ and CD8+ T cells in protective immunity to the murine nematode parasite *Trichuris muris*. Parasite Immunol. (1995) 17:161–5. 10.1111/j.1365-3024.1995.tb01018.x 7792100

[17] Else KJ Grencis RK. Antibody-independent effector mechanisms in resistance to the intestinal nematode parasite *Trichuris muris*. Infect Immun. (1996) 64:2950–4. 8757819 10.1128/iai.64.8.2950-2954.1996 PMC174173

[18] Blackwell NM Else KJ. B cells and antibodies are required for resistance to the parasitic gastrointestinal nematode *Trichuris muris*. Infect immun. (2001) 69:3860–8. 10.1128/IAI.69.6.3860-3868.2001 11349052 PMC98409

[19] Fu Y-X Chaplin DD. Development and maturation of secondary lymphoid tissues. Ann Rev Immunol. (1999) 17:399–433. 10.1146/annurev.immunol.17.1.399 10358764

[20] Golovkina TV Shlomchik M Hannum L Chervonsky A. Organogenic role of B lymphocytes in mucosal immunity. Science. (1999) 286:1965–8. 10.1126/science.286.5446.1965 10583962

[21] Baumgarth N Jager GC Herman OC Herzenberg LA Herzenberg LA. CD4+ T cells derived from B cell-deficient mice inhibit the establishment of peripheral B cell pools. Proc Natl Acad Sci USA. (2000) 97:4766–71. 10.1073/pnas.97.9.4766 10781082 PMC18307

[22] Sahputra R Yam-Puc JC Waisman A Muller W Else KJ. Evaluating the IgMi mouse as a novel tool to study B cell biology. Eur J Immunol. (2018) 48:2068–71. 10.1002/eji.201847735 30315705 PMC6750126

[23] deSchoolmeester ML Little MC Rollins BJ Else KJ. Absence of CC chemokine ligand 2 results in an altered Th1/Th2 cytokine balance and failure to expel *Trichuris muris* infection. J Immunol. (2003) 170:4693–700. 10.4049/jimmunol.170.9.4693 12707348

[24] Forman RA deSchoolmeester ML Hurst RJ Wright SH Pemberton AD Else KJ. The goblet cell is the cellular source of the anti-microbial angiogenin 4 in the large intestine post *Trichuris muris* infection. PLoS ONE. (2012) 7:e42248. 10.1371/journal.pone.0042248 22970115 PMC3435386

[25] Little MC Hurst RJ Else KJ. Dynamic changes in macrophage activation and proliferation during the development and resolution of intestinal inflammation. J Immunol. (2014) 193:4684–95. 10.4049/jimmunol.1400502 25261482 PMC4201944

[26] Zaretsky AG Taylor JJ King IL Marshall FA Mohrs M Pearce EJ T follicular helper cells differentiate from Th2 cells in response to helminth antigens. J Exp Med. (2009) 206:991–9. 10.1084/jem.20090303 19380637 PMC2715032

[27] Bancroft AJ McKenzie AN Grencis RK. A critical role for IL-13 in resistance to intestinal nematode infection. J Immunol. (1998) 160:3453–61. 9531306

[28] Else K Grencis R Cellular immune responses to the murine nematode parasite *Trichuris muris*. I. Differential cytokine production during acute or chronic infection. Immunology. (1991) 72:508. 1903765 PMC1384369

[29] Bancroft AJ Else KJ Sypek JP Grencis RK. Interleukin-12 promotes a chronic intestinal nematode infection. Eur J Immunol. (1997) 27:866–70. 10.1002/eji.1830270410 9130637

[30] Hasnain SZ Evans CM Roy M Gallagher AL Kindrachuk KN Barron L. Muc5ac: a critical component mediating the rejection of enteric nematodes. J Exp Med. (2011) 208:893–900. 10.1084/jem.20102057 21502330 PMC3092342

[31] Cruickshank SM Deschoolmeester ML Svensson M Howell G Bazakou A Logunova L. Rapid dendritic cell mobilization to the large intestinal epithelium is associated with resistance to Trichuris muris infection. J Immunol. (2009) 182:3055–62. 10.4049/jimmunol.0802749 19234202 PMC2671799

[32] Else K Finkelman F Maliszewski C Grencis R. Cytokine-mediated regulation of chronic intestinal helminth infection. J Exp Med. (1994) 179:347–51. 10.1084/jem.179.1.347 8270879 PMC2191309

[33] Else K Entwistle G Grencis R. Correlations between worm burden and markers of Th1 and Th2 cell subset induction in an inbred strain of mouse infected with *Trichuris muris*. Parasite Immunol. (1993) 15:595–600. 10.1111/pim.1993.15.10.595 7877836

[34] Else K Wakelin D. The effects of H-2 and non-H-2 genes on the expulsion of the nematode *Trichuris muris* from inbred and congenic mice. Parasitology. (1988) 96:543–50. 10.1017/S0031182000080173 3136419

[35] Uchida J Hamaguchi Y Oliver JA Ravetch JV Poe JC Haas KM. The innate mononuclear phagocyte network depletes B lymphocytes through Fc receptor–dependent mechanisms during anti-CD20 antibody immunotherapy. J Exp Med. (2004) 199:1659–69. 10.1084/jem.20040119 15210744 PMC2212805

[36] Tedder T Klejman G Disteche C Adler D Schlossman S Saito H. Cloning of a complementary DNA encoding a new mouse B lymphocyte differentiation antigen, homologous to the human B1 (CD20) antigen, and localization of the gene to chromosome 19. J Immunol. (1988) 141:4388–94. 2461992

[37] Tedder TF Forsgren A Boyd AW Nadler LM Schlossman SF Antibodies reactive with the B1 molecule inhibit cell cycle progression but not activation of human B lymphocytes. Eur J Immunol. (1986) 16:881–7. 10.1002/eji.1830160802 3091375

[38] Sahoo A Alekseev A Tanaka K Obertas L Lerman B Haymaker C. Batf is important for IL-4 expression in T follicular helper cells. Nat Commun. (2015) 6:7997. 10.1038/ncomms8997 26278622 PMC4557271

[39] Ait-Oufella H Herbin O Bouaziz J-D Binder CJ Uyttenhove C Laurans L. B cell depletion reduces the development of atherosclerosis in mice. J Exp Med. (2010) 207:1579–87. 10.1084/jem.20100155 20603314 PMC2916123

[40] León B Ballesteros-Tato A Browning JL Dunn R Randall TD Lund FE Regulation of TH2 development by CXCR5+ dendritic cells and lymphotoxin-expressing B cells. Nat Immunol. (2012) 13:681–90. 10.1038/ni.2309 22634865 PMC3548431

[41] Johansson-Lindbom B Borrebaeck CA. Germinal center B cells constitute a predominant physiological source of IL-4: implication for Th2 development *in vivo*. J Immunol. (2002) 168:3165–72. 10.4049/jimmunol.168.7.3165 11907068

[42] Appleton J McGregor D. Characterization of the immune mediator of rapid expulsion of *Trichinella spiralis* in suckling rats. Immunology. (1987) 62:477. 3499383 PMC1454122

[43] Harris N Pleass R Behnke J. Understanding the role of antibodies in murine infections with Heligmosomoides (polygyrus) bakeri: 35 years ago, now and 35 years ahead. Parasite Immunol. (2014) 36:115–24. 10.1111/pim.12057 23889357

[44] Pleass RJ Behnke JM. B-cells get the T-cells but antibodies get the worms. Trends Parasitol. (2009) 25:443–6. 10.1016/j.pt.2009.07.001 19734092 PMC3115547

[45] McCoy KD Stoel M Stettler R Merky P Fink K Senn BM. Polyclonal and specific antibodies mediate protective immunity against enteric helminth infection. Cell Host Microbe. (2008) 4:362–73. 10.1016/j.chom.2008.08.014 18854240

[46] Wojciechowski W Harris DP Sprague F Mousseau B Makris M Kusser K. Cytokine-producing effector B cells regulate type 2 immunity to H. polygyrus. Immunity. (2009) 30:421–33. 10.1016/j.immuni.2009.01.006 19249230 PMC2745290

[47] Liu Q Liu Z Rozo CT Hamed HA Alem F Urban JF. The role of B cells in the development of CD4 effector T cells during a polarized Th2 immune response. J Immunol. (2007) 179:3821–30. 10.4049/jimmunol.179.6.3821 17785819 PMC2258088

[48] Harris DP Goodrich S Mohrs K Mohrs M Lund FE. Cutting edge: the development of IL-4-producing B cells (B effector 2 cells) is controlled by IL-4, IL-4 receptor α, and Th2 cells. J Immunol. (2005) 175:7103–7. 10.4049/jimmunol.175.11.7103 16301612

[49] Lund FE. Cytokine-producing B lymphocytes—key regulators of immunity. Curr Opin Immunol. (2008) 20:332–8. 10.1016/j.coi.2008.03.003 18417336 PMC2474694

[50] Linton P-J Bautista B Biederman E Bradley ES Harbertson J Kondrack RM. Costimulation via OX40L expressed by B cells is sufficient to determine the extent of primary CD4 cell expansion and Th2 cytokine secretion *in vivo*. J Exp Med. (2003) 197:875–83. 10.1084/jem.20021290 12668647 PMC2193894

[51] Lu P Urban JF Zhou X Chen S Madden K Moorman M CD40-mediated stimulation contributes to lymphocyte proliferation, antibody production, eosinophilia, and mastocytosis during an *in vivo* type 2 response, but is not required for T cell IL-4 production. J Immunol. (1996) 156:3327–33. 8617957

[52] Liu Q Kreider T Bowdridge S Liu Z Song Y Gaydo AG. B cells have distinct roles in host protection against different nematode parasites. J Immunol. (2010) 184:5213–23. 10.4049/jimmunol.0902879 20357259 PMC3729113

[53] Ligas JA Kerepesi LA Galioto AM Lustigman S Nolan TJ Schad GA. Specificity and mechanism of immunoglobulin M (IgM)-and IgG-dependent protective immunity to larval Strongyloides stercoralis in mice. Infect Immun. (2003) 71:6835–43. 10.1128/IAI.71.12.6835-6843.2003 14638770 PMC308934

[54] Betts CJ Else KJ Mast cells, eosinophils and antibody-mediated cellular cytotoxicity are not critical in resistance to *Trichuris muris*. Parasite Immunol. (1999) 21:45–52. 10.1046/j.1365-3024.1999.00200.x 10081771

[55] Rajan B Ramalingam T Rajan TV. Critical role for IgM in host protection in experimental filarial infection. J Immunol. (2005) 175:1827–33. 10.4049/jimmunol.175.3.1827 16034125

[56] Inaba T Sato H Kamiya H. Monoclonal IgA antibody-mediated expulsion of Trichinella from the intestine of mice. Parasitology. (2003) 126:591–8. 10.1017/S003118200300310X 12866798

[57] Harris N Gause WC To B or not to B: B cells and the Th2-type immune response to helminths. Trends Immunol. (2011) 32:80–8. 10.1016/j.it.2010.11.005 21159556 PMC3076625

[58] Sahputra R Ruckerl D Couper K Muller W Else K The essential role played by B cells in supporting protective immunity against *Trichuris muris* infection is dependent on host genetic background and is independent of antibody. BioRxiv. (2019) 2019:550434 10.1101/550434 PMC6915098 31921120

