# Supplementary material for: The Essential Role Played by B Cells in Supporting Protective Immunity Against Trichuris muris Infection Is by Controlling the Th1/Th2 Balance in the Mesenteric Lymph Nodes and Depends on Host Genetic Background
Source: Front Immunol. 2019 Dec 10;10:2842. doi: 10.3389/fimmu.2019.02842 (PMC6915098; doi:10.3389/fimmu.2019.02842)
Supplement: Supplementary file 1 [file Presentation_1.pptx]

## Slide 1
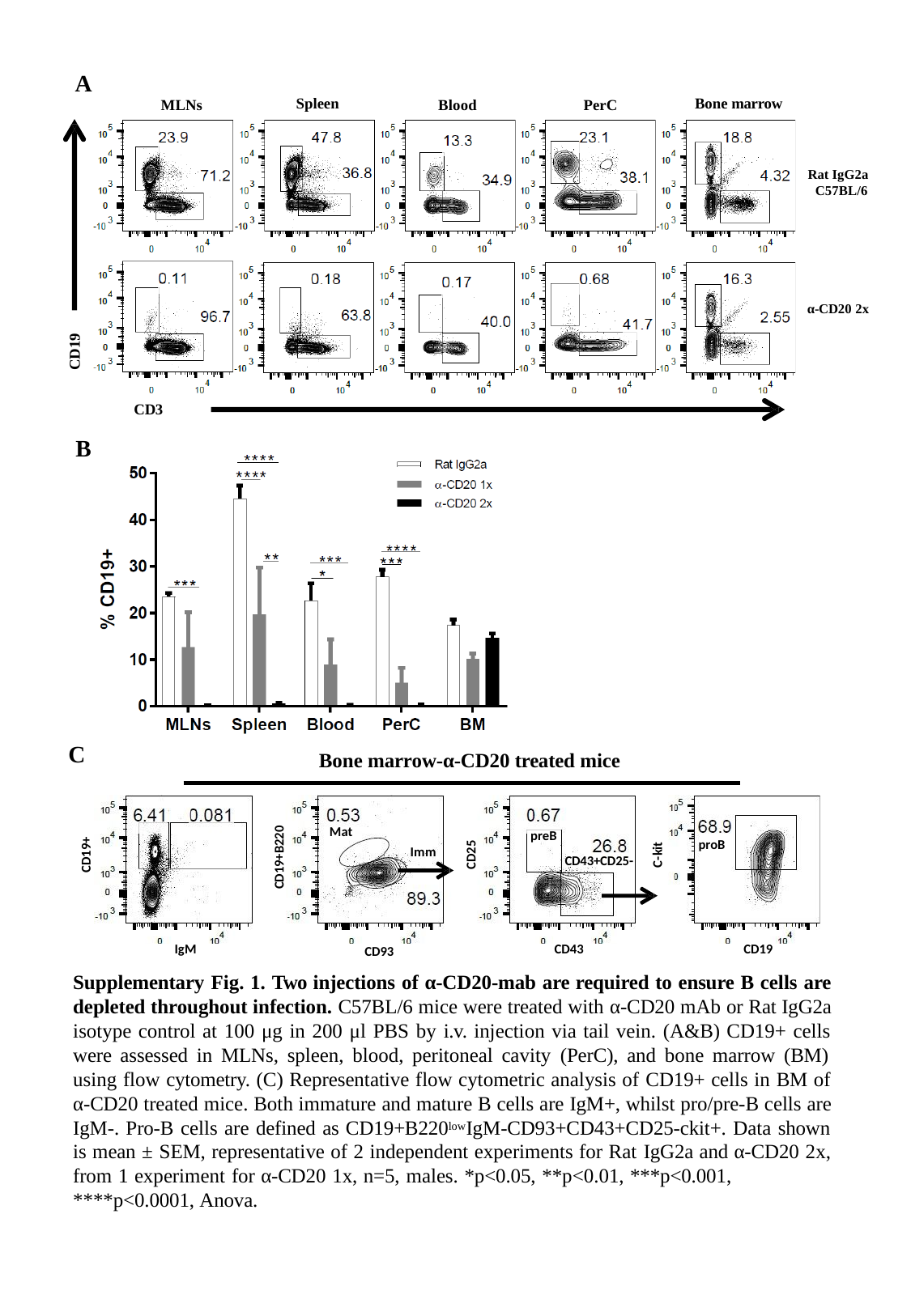

A
Spleen
Bone marrow
Blood
MLNs
PerC
Rat IgG2a C57BL/6
α-CD20 2x
CD19
CD3
B
C
Bone marrow-α-CD20 treated mice
Mat
CD19+B220
preB
proB
CD19+
CD25
C-kit
Imm
CD43+CD25-
IgM	CD43
CD19
CD93
Supplementary Fig. 1. Two injections of α-CD20-mab are required to ensure B cells are depleted throughout infection. C57BL/6 mice were treated with α-CD20 mAb or Rat IgG2a isotype control at 100 μg in 200 μl PBS by i.v. injection via tail vein. (A&B) CD19+ cells were assessed in MLNs, spleen, blood, peritoneal cavity (PerC), and bone marrow (BM) using flow cytometry. (C) Representative flow cytometric analysis of CD19+ cells in BM of α-CD20 treated mice. Both immature and mature B cells are IgM+, whilst pro/pre-B cells are IgM-. Pro-B cells are defined as CD19+B220lowIgM-CD93+CD43+CD25-ckit+. Data shown is mean ± SEM, representative of 2 independent experiments for Rat IgG2a and α-CD20 2x, from 1 experiment for α-CD20 1x, n=5, males. *p<0.05, **p<0.01, ***p<0.001,
****p<0.0001, Anova.

## Slide 2
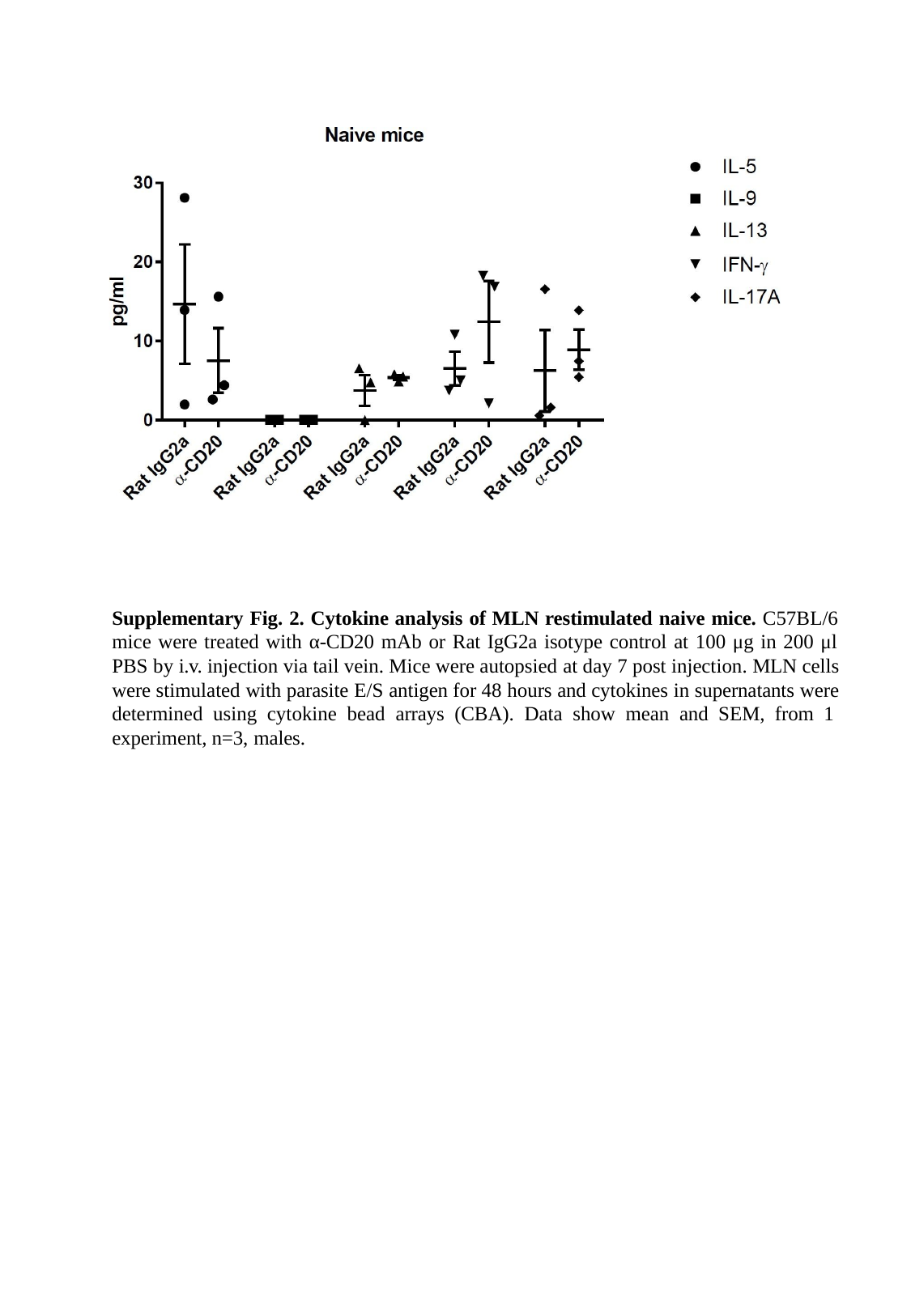

Supplementary Fig. 2. Cytokine analysis of MLN restimulated naive mice. C57BL/6 mice were treated with α-CD20 mAb or Rat IgG2a isotype control at 100 μg in 200 μl PBS by i.v. injection via tail vein. Mice were autopsied at day 7 post injection. MLN cells were stimulated with parasite E/S antigen for 48 hours and cytokines in supernatants were determined using cytokine bead arrays (CBA). Data show mean and SEM, from 1 experiment, n=3, males.

## Slide 3
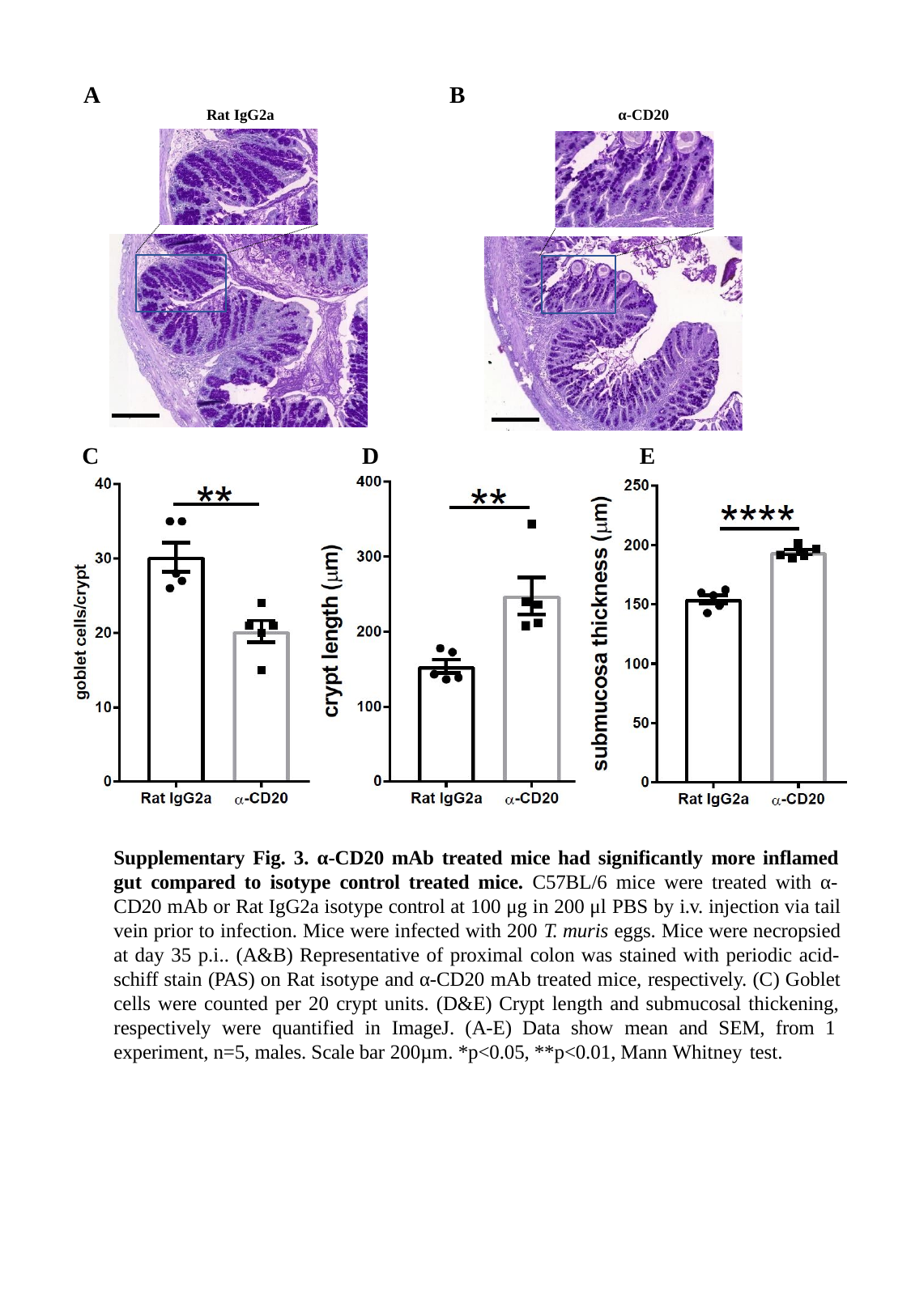

A
B
Rat IgG2a
α-CD20
C
D
E
Supplementary Fig. 3. α-CD20 mAb treated mice had significantly more inflamed gut compared to isotype control treated mice. C57BL/6 mice were treated with α- CD20 mAb or Rat IgG2a isotype control at 100 μg in 200 μl PBS by i.v. injection via tail vein prior to infection. Mice were infected with 200 T. muris eggs. Mice were necropsied at day 35 p.i.. (A&B) Representative of proximal colon was stained with periodic acid- schiff stain (PAS) on Rat isotype and α-CD20 mAb treated mice, respectively. (C) Goblet cells were counted per 20 crypt units. (D&E) Crypt length and submucosal thickening, respectively were quantified in ImageJ. (A-E) Data show mean and SEM, from 1 experiment, n=5, males. Scale bar 200µm. *p<0.05, **p<0.01, Mann Whitney test.
